# ACTIVE USE OF ONLINE COMMUNITY REDUCES LONELINESS AMONG OLDER ADULTS THROUGH WEAK TIES FORMATION

**DOI:** 10.1093/geroni/igad104.2719

**Published:** 2023-12-21

**Authors:** Nahyun Kim, Keiko Katagiri

**Affiliations:** Kobe University, Kobe, Hyogo, Japan; Kobe University, Kobe, Hyogo, Japan

## Abstract

Online communities could aid older adults in expanding their social connections and developing weak ties, which may help alleviate their loneliness. However, research investigating the impact of online community participation on the formation of weak ties among older adults remains lacking. This study aimed to investigate 1) the relationship between active use of an online community designed for older adults and the formation of weak ties within the platform, and 2) the potential for these weak ties to alleviate loneliness among members. The online community platform for older adults allowed users to post diary entries and photos, join interest-based communities, and organize virtual and in-person events. An online survey was conducted among members aged 60 to 79 years (N=862). The survey measured active use, weak ties (measured by the number of mutual followers), and loneliness. Path analysis showed that active engagement in the online community was positively associated with the formation of weak ties, which, in turn, were negatively associated with loneliness. Thus, older adults who frequently posted own content and participated in community activities were more likely to form many weak ties in the platform, and these weak ties may help reduce their loneliness. However, this study only examined online community use, meaning that further research is needed to explore the relationship between general social networking services use and weak ties, as well as examine the context of the passive use of these services.

